# Whole-Genome Shotgun Sequence of Bacillus mycoides Strain U53, a Psychrotolerant Bacterium Isolated from the Sakhalin Region in Russia

**DOI:** 10.1128/MRA.01257-20

**Published:** 2021-01-07

**Authors:** Ha My T. Phan, Marina L. Sidorenko, Po-Chun Tsai, Shir-Ly Huang

**Affiliations:** a Institute of Microbiology and Immunology, National Yang-Ming University, Taipei, Taiwan; b Federal Scientific Center of the East Asia Terrestrial Biodiversity, Far East Branch of the Russian Academy of Sciences, Vladivostok, Russia; University of Rochester School of Medicine and Dentistry

## Abstract

The shotgun genome sequence of Bacillus mycoides strain U53, a psychrotolerant bacterium that was isolated from soil in Russia, is characterized. The bacterial genome is 5,710,017 bp long and is predicted to have 5,847 genes. The GC content is 35.4%.

## ANNOUNCEMENT

Bacillus mycoides is a soil-dwelling, Gram-positive, spore-forming bacterium belonging to the group of Bacillus cereus sensu lato (1, 2). Because of their abundance in soil, heat resistance (forming spores), and potential to grow in food products at low temperature (3), psychrotolerant B. mycoides strains have been of concern for the food industry (4–7). The study of psychrotolerant microbes provides an understanding of the cold adaptation lifestyle.

Strain U53 was isolated from soil in the Sakhalin region in Russia. After removal of 1- to 2-cm depth of surface soil, 30 g soil was collected; 1 g soil was then suspended in 0.9% NaCl and shaken for 2 h at 15°C. The soil suspension was spread onto peptone agar plates (1% peptone, 0.5% NaCl, and 2% agar [pH 7.4]) and incubated at 4°C for 15 days. Strain U53 was further shown to be able to grow at 4°C to 37°C on nutrient agar plates.

A mid-log-phase culture of strain U53 grown in nutrient broth at 30°C at 200 rpm was harvested. Genomic DNA was extracted by the phenol-chloroform method (8). After PCR using the 16S rRNA gene universal primers 27F (5′-AGAGTTTGATCCTGGCTCAG-3′) and 1492R (5′-GGTTACCTTGTTACGACTT-3′), the amplified sequence was used for bacterial identification. The BLAST results from the NCBI (https://blast.ncbi.nlm.nih.gov/Blast.cgi) showed that the 16S rRNA gene sequence of strain U53 is completely identical to that of B. mycoides strain ATCC 6462^T^ but with different morphology under a light microscope. For genome sequencing, DNA quality was verified by the Qubit double-stranded DNA (dsDNA) high-sensitivity (HS) assay, followed by Oxford Nanopore Technologies sequencing on the GridION X5 platform with a FLO-MIN106D flow cell at Health GeneTech Corp. (Taoyuan, Taiwan). The genome library was analyzed using the ligation sequencing kit 1D (Oxford Nanopore Technologies) according to the manufacturer's instructions; the DNA was not sheared or size selected. All raw data (fast5 format) were base called by Guppy v3.4 (https://github.com/nanoporetech/pyguppyclient) to obtain the raw fastq files, which were further demultiplexed with qcat v1.0.6 (https://github.com/nanoporetech/qcat), statistically analyzed, and plotted with NanoPlot 1.0.0 (9). A total of 349,449 reads, with a mean read length of 10,162 bp and an *N*_50_ value of 16,222 bp, were generated. After read cleaning, trimming, and filtering by NanoFilt v2.5.0 (9), the cleaned fastq files were mapped using minimap2 (10). Read mapping was performed against a reference database, using the B. mycoides strain ATCC 6462 sequence (GenBank accession number NZ_CP009692.1). These mapped reads were then corrected with Canu v2.0 (11). *De novo* assembly was performed using Flye v2.7.1 (12) with a polishing assembly using minimap2 and Racon v1.4.3 (13) to obtain the draft genome. Default parameters were used for all software unless otherwise specified.

The assembled genome of B. mycoides U53 contains four contigs with a total of 5,710,017 bp (GC content of 35.4%) and an *N*_50_ value of 5,601,634 bp. All assembled contigs were annotated using PGAP v4.11 (14), and 5,847 genes, including 153 RNA genes, were identified.

### Data availability.

This whole-genome shotgun project was deposited in GenBank (accession number JABURZ000000000), and the raw reads were deposited in the NCBI SRA (accession number SRR12077163). The version described in this paper is the first version, JABURZ000000000.1.
